# Additional postdilatation using noncompliant balloons after everolimus‐eluting stent implantation: Results of the PRESS trial

**DOI:** 10.1002/clc.23355

**Published:** 2020-03-16

**Authors:** Gyung‐Min Park, Jae‐Hwan Lee, Si Wan Choi, Jin‐Ok Jeong, Eun‐Seok Shin, Jang‐Whan Bae, Hyuck‐Jun Yoon, Kyung Tae Jung, Ju Yeol Baek, Woong Gil Choi, Rak Kyeong Choi, Sung‐Ho Her, Jin Bae Lee, Jon Suh, Jae Beom Lee, Se‐Whan Lee, In‐Ho Chae, So‐Yeon Choi, In‐Whan Seong

**Affiliations:** ^1^ Department of Cardiology Ulsan University Hospital, University of Ulsan College of Medicine Ulsan South Korea; ^2^ Department of Cardiology Chungnam National University Hospital, Chungnam National University School of Medicine Daejeon South Korea; ^3^ Department of Internal Medicine Chungbuk National University College of Medicine Cheongju South Korea; ^4^ Department of Cardiology Keimyung University Dongsan Medical Center Daegu South Korea; ^5^ Department of Cardiology Eulji University Hospital Daejeon South Korea; ^6^ Department of Cardiology Cheongju St. Mary's Hospital Cheongju South Korea; ^7^ Department of Internal Medicine, School of Medicine Konkuk University Chungju South Korea; ^8^ Department of Cardiology Mediplex Sejong Hospital Bucheon South Korea; ^9^ Department of Cardiology Daejeon St. Mary's Hospital, The Catholic University of Korea Seoul South Korea; ^10^ Department of Cardiology Daegu Catholic University Medical Center Daegu South Korea; ^11^ Department of Cardiology Soonchunhyang University Bucheon Hospital Bucheon South Korea; ^12^ Department of Cardiology Anyang Sam Hospital Anyang South Korea; ^13^ Department of Cardiology Soonchunhyang University Cheonan Hospital Cheonan South Korea; ^14^ Department of Cardiology Seoul National University Bundang Hospital Bundang South Korea; ^15^ Department of Cardiology Ajou University Hospital Suwon South Korea

**Keywords:** drug‐eluting stent, everolimus‐eluting stent, noncompliant balloon, postdilatation

## Abstract

**Background:**

There are limited data on the clinical value of routine postdilatation using noncompliant balloons after contemporary drug‐eluting stent implantation.

**Hypothesis:**

Additional postdilatation using noncompliant balloons after everolimus‐eluting stent implantation could provide better clinical outcomes.

**Methods:**

We randomly assigned 1774 patients with coronary artery disease to undergo additional high‐pressure postdilatation using noncompliant balloons and moderate‐pressure dilatation using stent balloons after everolimus‐eluting stent implantation. The primary endpoint was a composite of death, myocardial infarction (MI), stent thrombosis, and target vessel revascularization (TVR) 2 years after randomization.

**Results:**

The study was discontinued early owing to slow enrollment. In total, 810 patients (406 patients in the high pressure group and 404 in the moderate pressure group) were finally enrolled. At 2 years, the primary endpoint occurred in 3.6% of patients in the high pressure group and in 4.4% of those in the moderate pressure group (*P* = .537). In addition, no significant differences were observed between the two groups in the occurrence of an individual end point of death (0.8% in the high pressure group vs 1.5% in the moderate group, *P* = .304), MI (0.2% vs 0.5%, *P* = .554), stent thrombosis (0% vs 0.2%, *P* = .316), or TVR (2.8% vs 2.6%, *P* = .880).

**Conclusions:**

The strategy of routine postdilatation using noncompliant balloons after everolimus‐eluting stent implantation did not provide incremental clinical benefits.

## 1 INTRODUCTION

Optimization of stent deployment during percutaneous coronary intervention (PCI) is a key element for improving clinical outcomes.^1^ With the advent of drug‐eluting stents (DESs), DESs have become the main strategy for PCI because they have significantly reduced the need for repeat revascularization.^2^, ^3^ However, suboptimal stent deployment frequently occurs during DES implantation, which may increase the risk of in‐stent restenosis and stent thrombosis.^4^, ^5^, ^6^, ^7^, ^8^ Therefore, even in the DES era, optimal deployment of stents remains a challenging issue.

In bare‐metal stent, adjunctive postdilatation using noncompliant balloons after stent implantation provided further stent optimization to reduce the incidence of in‐stent restenosis and stent thrombosis.^9^, ^10^ Even in the DES, previous studies also support the use of postdilatation with noncompliant balloons after deployment of DES.^11^, ^12^, ^13^ In addition, a recent randomized trial showed that stent optimization with intravascular ultrasound (IVUS) and adjunct postdilatation would be helpful in patients requiring long coronary stent implantation with a discrepancy in coronary artery diameter.^14^ However, there is still a lack of evidence from randomized trials to evaluate the clinical benefits of postdilatation using noncompliant balloons in patients undergoing contemporary DES implantation. Therefore, we aimed to investigate the clinical effect of postdilatation from the PRESS trial (the impact of additional high PRessure in‐stEnt dilatation uSing noncompliant balloons after Xience Stent implantation).

## 2 PATIENTS AND METHODS

### 2.1 Study design and population

This prospective, multicenter, open‐label, randomized controlled trial included 810 patients aged ≥18 years with coronary artery disease who underwent everolimus‐eluting stent (Xience prime, Abbott Vascular, Santa Clara, California) implantation. This study involved 15 cardiac centers in Korea between February 2012 and October 2015. Patients were considered eligible if they had either stable angina or acute coronary syndrome and had at least one de novo coronary lesion (defined as a visual vessel diameter of ≥2.5 mm, diameter stenosis of ≥50%, and lesion length of ≤70 mm, in which the lesion is covered with ≤2 stents) suitable for stent implantation. Patients were excluded if they had contraindications to aspirin and clopidogrel, unprotected left main disease (diameter stenosis of ≥50% by visual estimate), graft vessel disease, in‐stent restenotic lesion, bifurcation lesion requiring stent implantation for both main and side branches, history of bleeding diathesis or coagulopathy, hepatic dysfunction with aspartate aminotransferase or alanine aminotransferase level ≥3 times the upper normal reference limit, history of renal dysfunction or serum creatinine level of ≥2.0 mg/dL, serious noncardiac comorbid disease with a life expectancy of <2 years, ST‐elevation acute myocardial infarction (MI) within 2 weeks, planned major surgery within the next 6 months with the need to discontinue antiplatelet therapy, or inability to follow the protocol. In patients with multiple lesions who fulfilled the inclusion and exclusion criteria, the first stented lesion was considered the target lesion. The institutional review board at each participating center approved the protocol. All patients provided written informed consent.

### 2.2 Randomization and study procedures

Patients who met the inclusion and exclusion criteria were randomly assigned in a 1:1 ratio to receive additional high‐pressure postdilatation using a noncompliant balloon and moderate‐pressure dilatation using a stent balloon with an interactive web response system. The allocation sequence was computer generated and stratified according to participating center and blocked with block sizes of four and six varying randomly.

**Figure 1:**
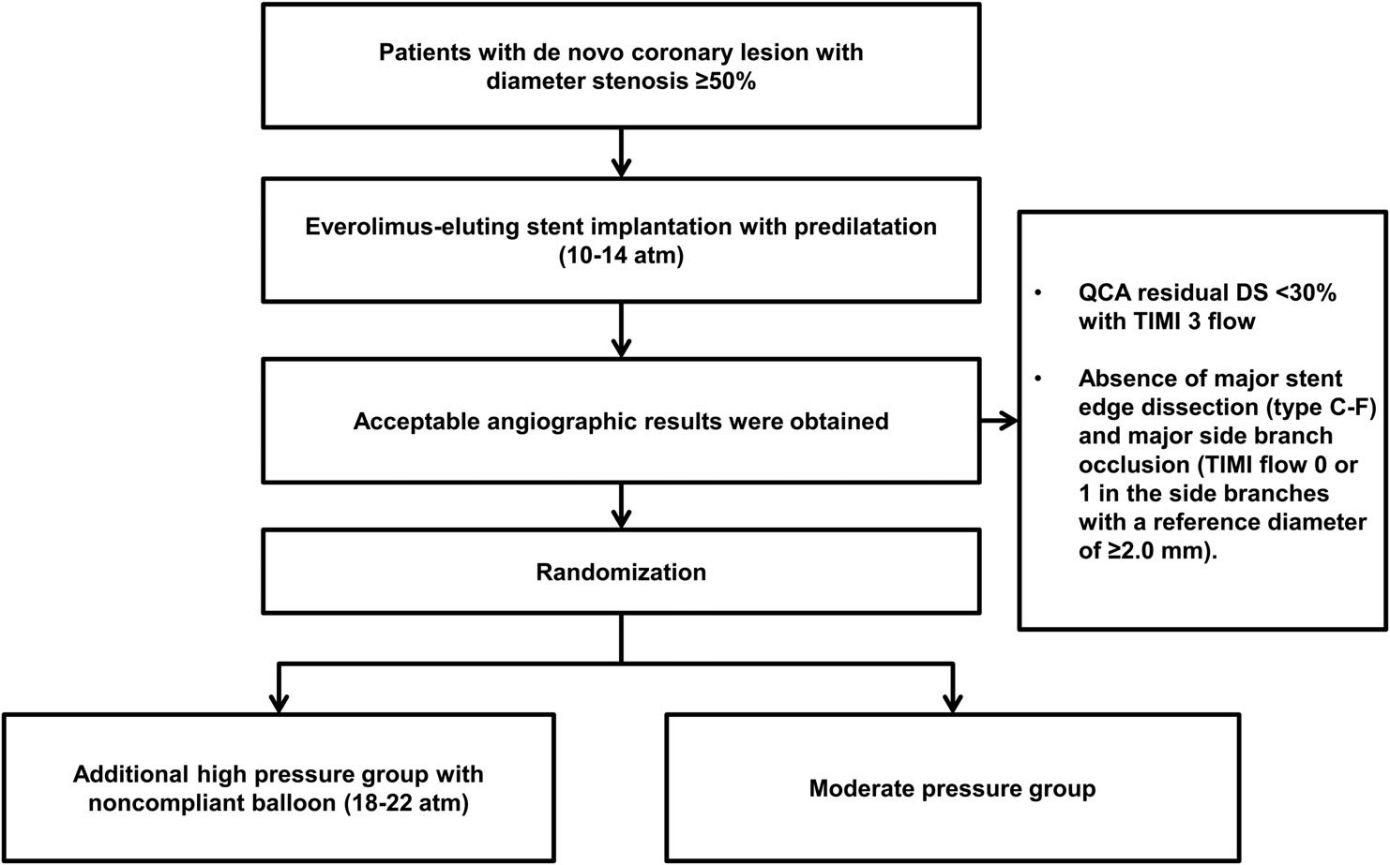
Study flow. DS, diameter stenosis; QCA, quantitative angiographic analysis

The procedure was performed using standard techniques. The stents were deployed at 10 to 14 atm with their stent balloon system. If an acceptable result was achieved, randomization was performed. An acceptable result was defined as a quantitative angiographic residual diameter stenosis of <30% with TIMI (Thrombolysis in Myocardial Infarction) grade 3 flow and absence of major stent edge dissection (type C‐F) and major side branch occlusion (TIMI flow 0 or 1 in the side branches with a reference diameter of ≥2.0 mm). In the moderate pressure group, the procedure was finished. In the high pressure group, postdilatation with at least the same or larger size noncompliant balloons at 18 to 22 atm was followed (Figure 1).

From at least 24 hours before the procedure and thereafter, all patients received aspirin (loading dose of 300 mg, followed by 100 mg/day indefinitely) and clopidogrel (loading dose of 300‐600 mg, followed by 75 mg/day for at least 12 months). Heparin was administered throughout the procedure to maintain an activated clotting time of ≥250 seconds. The use of IVUS and administration of glycoprotein IIb/IIIa inhibitors were at the discretion of the operator.

### 2.3 Study endpoint

The primary endpoint was defined as the occurrence of major adverse cardiac events, including all‐cause death, MI, stent thrombosis, and target vessel revascularization (TVR) 2 years after the procedure. The secondary endpoints included the occurrence of an individual endpoint of death, MI, stent thrombosis, and TVR at 2 years. At 2 years, a composite of death and MI was also assessed.

Death was considered to be cardiac in etiology unless an unequivocal noncardiac cause was established. MI was diagnosed on the basis of an increase in the creatine kinase MB (myocardial band) fraction or troponin level greater than the 99th percentile of the upper normal limit with at least one of the following aspects: ischemic symptoms, electrocardiographic changes, and abnormal imaging findings of MI.^15^, ^16^ Definite, probable, and possible stent thrombosis was defined according to the Academic Research Consortium.^16^ TVR was defined as any repeat revascularization of the target vessel with either of the following: (a) at least 50% of the diameter stenosis on quantitative coronary angiographic analysis with ischemic symptoms or a positive stress test finding or (b) at least 70% of the diameter stenosis on quantitative coronary angiographic analysis.

### 2.5 Statistical analysis

On the basis of results from previous studies,^17^, ^18^ we assumed a primary endpoint of 7% in the additional high pressure group and 10% in the moderate pressure group at the 2‐year follow‐up. Using a two‐sided 5% significance level, we estimated that 797 patients per group were needed to detect this difference with a statistical power of 80%. Considering a 5% follow‐up loss, total sample size was estimated to 1744 patients (872 patients per group). However, because of a much slower than anticipated enrollment, enrollment was stopped in October 2015 as recommended by the data and safety monitoring board, by which time 810 patients had been enrolled.

All analyses of the two groups were performed according to the intention‐to‐treat principle. Continuous variables are presented as means ± SD or medians (interquartile ranges) and compared using the *t* test or Mann‐Whitney test. Categorical variables were presented as numbers and percentages and compared using the *χ* ^2^ test or Fisher's exact test. Survival was assessed using the Kaplan‐Meier method and compared using the log‐rank test. In the patients with multiple clinical events, the first event was considered to be the component of composite outcome. Statistical analyses were performed using the time of first event from randomization. A *P* value of <.05 was considered statistically significant. All statistical analyses were performed using the SPSS software (version 18.0, SPSS Inc., Chicago, Illinois).

## 3 RESULTS

### 3.1 Study population

Between February 2012 and October 2015, 810 patients were randomly assigned to the high pressure group (n = 406) and moderate pressure group (n = 404) (Supplementary Figure 1). Among the 406 patients randomly assigned to the high pressure group, additional high‐pressure postdilatation using noncompliant balloons were not performed for two patients (0.5%) owing to the physician's decision. Conversely, among the 404 patients assigned to the moderate pressure group, two (0.5%) were treated with adjunctive noncompliant balloon postdilatation because of unfavorable calcification. The mean age of the study population was 61.8 ± 9.0 years, and 572 (70.6%) patients were men. The clinical presentations of the study participants were stable angina in 326 patients (40.2%), unstable angina in 378 (46.7%), and acute MI in 106 (13.1%). The baseline demographic and clinical characteristics of the study population were well balanced between the two groups (Table 1).

**Table 1:** Baseline characteristics of the study population

Baseline characteristics	High pressure (n = 406)	Moderate pressure (n = 404)	*P* value
Age, years	61.8 ± 9.0	61.8 ± 9.0	0.976
Men, no. (%)	289 (71.2)	283 (70.0)	0.723
Body mass index, kg/m^2^	25.0 ± 3.0	25.0 ± 3.1	0.997
Systolic blood pressure, mm Hg	138.0 ± 22.1	140.2 ± 24.0	0.169
Diastolic blood pressure, mm Hg	81.1 ± 12.8	80.6 ± 13.1	0.630
Hypertension, no. (%)	240 (59.4)	262 (65.0)	0.101
Diabetes mellitus, no. (%)	131 (32.3)	125 (31.0)	0.703
Insulin‐dependent diabetes mellitus, no. (%)	16 (12.9)	9 (7.5)	0.164
Hyperlipidemia, no. (%)	174 (43.2)	184 (45.9)	0.440
Current smoking, no. (%)	117 (30.0)	120 (30.8)	0.815
Previous myocardial infarction, no. (%)	10 (2.5)	9 (2.2)	0.829
Previous percutaneous coronary intervention, no. (%)	27 (6.7)	35 (8.7)	0.286
Prior coronary artery bypass graft, no. (%)	1 (0.2)	1 (0.2)	0.999
Left ventricular ejection fraction, %	62.6 ± 8.3	63.2 ± 8.2	0.317
Clinical presentation, no. (%)			0.328
Stable angina	153 (37.7)	173 (42.8)	
Unstable angina	198 (48.8)	180 (44.6)	
Acute myocardial infarction	55 (13.5)	51 (12.6)	
Number of diseased vessels, no. (%)			0.947
1	190 (46.8)	192 (47.5)	
2	130 (32.0)	125 (30.9)	
3	86 (21.2)	87 (21.5)	
Number of treated lesions per patient	1.3 ± 0.6	1.4 ± 0.7	0.265
Medications at discharge, no. (%)			
Aspirin	404 (99.8)	403 (99.8)	0.999
Clopidogrel	398 (98.3)	398 (98.5)	0.783
Statin	387 (95.6)	386 (95.5)	0.994
Beta‐blocker	267 (65.9)	259 (64.1)	0.588
ACEI/ARB	126 (31.1)	122 (30.2)	0.778

*Note*: Data are expressed as n (%) or means ± SD.

Abbreviations: ACEI, angiotensin‐converting‐enzyme inhibitor; ARB, angiotensin receptor blocker.

### 3.2 Angiographic and procedural characteristics

The lesion type B2 or C was seen in 568 patients (71%). However, moderate to severe calcification was observed in 44 patients (5.4%). Seventy‐seven patients (9.5%) were treated with overlapping stents. The mean stented length of the target lesions was 28.7 ± 12.6 mm. Although the mean final balloon size was similar between the two groups, the maximal inflation pressure was higher in the high pressure group than in the moderate pressure group (19.1 ± 2.6 atm vs 12.4 ± 2.1 atm, *P* < .001). Consequently, on postprocedural quantitative angiographic analysis, the in‐stent acute gain was higher and the in‐stent diameter stenosis was smaller in the high pressure group than in the moderate pressure group. However, the incidence of edge tear or perforation and no reflow or distal embolization was low and comparable between the two groups (Table 2).

**Table 2:** Angiographic and procedural characteristics of target lesions

	High pressure (n = 406)	Moderate pressure (n = 404)	*P* value
Target coronary artery, no. (%)			.356
Left anterior descending artery	220 (49.8)	221 (54.7)	
Left circumflex artery	83 (20.4)	77 (19.1)	
Right coronary artery	121 (29.8)	106 (26.2)	
Type B2 or C lesion, no. (%)	277 (68.2)	291 (72.0)	.237
Total occlusion, no. (%)	19 (4.7)	23 (5.7)	.510
Bifurcation lesions, no. (%)	72 (17.7)	68 (16.8)	.734
Moderate to severe calcification, no. (%)	25 (6.2)	19 (4.7)	.361
Use of intravascular ultrasound, no. (%)	223 (54.9)	223 (55.2)	.938
No reflow or distal embolization, no. (%)	3 (0.7)	3 (0.7)	.995
Edge dissection or coronary perforation, no. (%)	1 (0.2)	1 (0.2)	.999
Number of used stents at the target lesion	1.1 ± 0.3	1.1 ± 0.3	.812
Stented length, mm	28.3 ± 11.9	29.1 ± 13.3	.372
Maximal inflation pressure, atm	19.1 ± 2.6	12.4 ± 2.1	<.001
Largest balloon size, mm	3.4 ± 0.5	3.4 ± 0.9	.668
Baseline quantitative coronary angiographic data			
Reference vessel diameter, mm	2.76 ± 0.54	2.71 ± 0.54	.244
Minimum lumen diameter, mm	0.75 ± 0.48	0.77 ± 0.50	.595
Lesion length, mm	21.3 ± 10.9	21.3 ± 11.5	.979
Diameter stenosis, %	73.2 ± 15.8	72.4 ± 16.5	.504
Postprocedural quantitative coronary angiographic data			
Minimum lumen diameter, mm	2.16 ± 0.52	2.10 ± 0.50	.105
In‐stent diameter stenosis, %	4.3 ± 10.7	8.9 ± 11.6	<.001
In‐segment diameter stenosis, %	11.9 ± 10.6	13.3 ± 11.5	.112
In‐stent acute gain, mm	1.75 ± 0.61	1.60 ± 0.54	.001
In‐segment acute gain, mm	1.41 ± 0.65	1.33 ± 0.56	.066

*Note*: Data are expressed as n (%) or means ± SD.

### 3.3 Clinical outcomes

**Figure 2:**
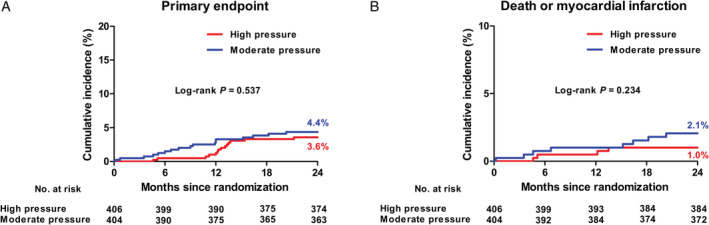
Kaplan‐Meier curves at 2 years. A, The cumulative incidence of the primary composite endpoint of all‐cause death, myocardial infarction, stent thrombosis, and target vascular revascularization. B, The cumulative incidence of the composite endpoint of all‐cause death and myocardial infarction. The event rates are shown as Kaplan‐Meier estimates. The *P* value was calculated using the log‐rank test

Table 3 shows the clinical outcomes. At 2 years, the primary endpoint occurred in 3.6% of patients in the high pressure group and in 4.4% of those in the moderate pressure group (*P* = .537) (Figure 2A). In addition, there were no significant differences between the two groups in the occurrence of an individual end point of death (0.8% in the high pressure group vs 1.5% in the moderate group, *P* = .304), MI (0.2% vs 0.5%, *P* = .554), stent thrombosis (0% vs 0.2%, *P* = .316), TVR (2.8% vs 2.6%, *P* = .880), and death/MI (1.0% vs 2.1%, *P* = .234) (Figure 2B).

**Table 3:** Clinical outcomes over 2 years

	High pressure (n = 406)	Moderate pressure (n = 404)	*P* value*
Primary endpoint, no. (%)			
All‐cause death/myocardial infarction/stent thrombosis/target vessel revascularization	14 (3.6)	17 (4.4)	.537
Secondary endpoint, no. (%)			
Death	3 (0.8)	6 (1.5)	.304
Cardiac	2 (0.5)	4 (1.0)	
Noncardiac	1 (0.2)	2 (0.5)	
Myocardial infarction	1 (0.2)	2 (0.5)	.554
Stent thrombosis	0 (0)	1 (0.2)	.316
Target vessel revascularization	11 (2.8)	10 (2.6)	.880
All‐case death/myocardial infarction	4 (1.0)	8 (2.1)	.234

*Note*: Values are presented as n (%) as determined using the Kaplan‐Meier method. *P‐values were calculated using the log‐rank test.

## 4 DISCUSSION

The main findings of this study were as follows: (a) in the patients who underwent contemporary everolimus‐eluting stent implantation, postdilatation using noncompliant balloons was safely performed without increasing incidence of adverse events; (b) postdilatation resulted in higher in‐stent acute gain and smaller in‐stent diameter stenosis; (c) however, the routine postdilatation strategy did not improve the 2‐year clinical outcomes; and (d) therefore, the routine postdilatation approach should be carefully considered in contemporary DES implantation.

Optimal stent deployment during PCI has an important effect on short‐ and long‐term clinical outcomes.^1^ There has been significant advances in platforms, drugs, and polymers in DESs. However, the incidence of suboptimal stent deployment was up to 30% even in the DES era.^4^ In previous several reports, postdilatation of DES, improving minimal stent area and minimal stent diameter, showed improved clinical outcomes, but they analyzed limited populations in registries data and did not focus on the contemporary DES.^11^, ^12^, ^13^ On the other hand, contemporary everolimus‐eluting stents demonstrated better clinical efficacy and safety compared with other DESs or bare‐metal stents.^17^, ^19^, ^20^ However, there is still a lack of evidence as to whether postdilatation using noncompliant balloons at high pressures following deployment of the contemporary DESs is associated with an improvement in clinical outcomes. Therefore, to address the clinical usefulness of high‐pressure noncompliant postdilatation after contemporary everolimus‐eluting stent implantation, the present study was designed.

While reducing the risk of in‐stent restenosis and stent thrombosis, there are potential adverse effects from postdilatation. Postdilatation using noncompliant balloons at high pressures could be associated with a risk of edge tear and vessel rupture.^21^, ^22^ There was also some evidence that aggressive stent expansion with postdilatation could lead to distal embolization and an increased incidence of periprocedural MI.^23^, ^24^ However, in the present study, the incidence of edge dissection or coronary perforation between the high and low pressure groups was much low and comparable. In addition, although our study included 484 patients (59.8%) with acute coronary syndrome, there were no significant differences in the occurrence of no reflow or distal embolization between the two groups. Therefore, these findings indicate that postdilatation using noncompliant balloons could be performed safely during PCI.

In the bare‐metal stent era, deployment of such stents was often associated with suboptimal stent expansion.^21^ Adjunctive postdilatation using noncompliant balloons improved stent expansion and reduced in‐stent restenosis and stent thrombosis.^9^, ^10^, ^21^ Even in the DES era, these suboptimal stent expansions were also reported in the literature in association with an increased risk of in‐stent restenosis and repeat revascularization rates and might also predispose to stent thrombosis.^6^, ^7^, ^8^ Similar to a previous study,^11^ this study showed that postdilatation resulted in higher in‐stent acute gain and smaller in‐stent diameter stenosis. However, our study failed to demonstrate the clinical advantages of routine postdilatation. Therefore, in the majority of patients undergoing contemporary DES implantation, routine postdilatation might not be mandatory if DES deployment is successfully performed.

In our study, there are possibilities why the routine strategy of postdilatation using noncompliant balloons did not provide incremental clinical benefits. Complex lesion subsets, such as heavy calcification, large plaque burden, small vessel, in‐stent restenosis, or long lesion, are associated with a lower success rate and require more attention and specialized devices to obtain an optimal stent deployment.^1^ In a recent large randomized study (n = 1400, mean stented length 39.3 mm), stent optimization with IVUS and adjunct postdilatation was significantly associated with improved clinical outcomes in patients requiring long coronary stent implantation.^14^ However, in a small randomized trial with shorter stent lengths (n = 543, mean stented length 32.3 mm), IVUS‐guided PCI with adjunct postdilatation did not show clinical benefits compared with angiography‐guided PCI.^25^ In the present study, although IVUS was used in 446 patients (55.1%), we enrolled patients with relatively fewer complex lesions and the mean stented length was shorter (28.7 mm). Accordingly, the routine strategy of postdilatation using noncompliant balloons after everolimus‐eluting stent implantation would not provide clinical benefits at 2 years. In addition, postdilatation might be more clinically helpful for patients at high‐risk for in‐stent restenosis and stent thrombosis, such as those with diabetes mellitus, low ejection fraction, and renal failure. Therefore, further studies are required to elucidate the clinical usefulness of postdilatation using noncompliant balloons in these complex lesion subsets and high‐risk patients.

Our study has also several limitations. First, in South Korea, the registration of clinical studies was not mandatory until March 2018. Since the present study has been conducted in 15 cardiac centers of South Korea between February 2012 and October 2015, our study was possible by approvals of the institutional review board at each participating center without legal restrictions. Second, the decision of enrollment of study was made by the attending operators. Unfortunately, we have no reliable data for patients screened and excluded from the current study. Third, the overall clinical event rate was lower than anticipated. Moreover, the present study was discontinued early due to slow enrollment. Therefore, the sample size could be insufficient for evaluating whether the routine postdilatation strategy benefited all subgroups. Fourth, the use of IVUS was at the discretion of operating physicians. In previous and our studies, routine use of IVUS or postdilatation during PCI did not show incremental clinical benefits.^26^ However, as shown a recent randomized study,^14^ selective postdilatation with specific IVUS criteria for optimal stent optimization might improve clinical outcomes in specific populations. Fifth, although postdilatation achieved higher in‐stent acute gain and smaller in‐stent diameter stenosis, our study did not address the long‐term clinical benefits beyond the 2‐year follow‐up.

In conclusion, the strategy of routine application of postdilatation using noncompliant balloons after everolimus‐eluting stent implantation did not improve clinical outcomes at 2 years. However, the present study did not have enough statistical power to evaluate the clinical effects of additional postdilatation. Therefore, these findings should be confirmed in further randomized clinical trials with larger populations.

## CONFLICT OF INTEREST

The authors declare no potential conflict of interests.

## Supporting information

Supplementary Figure 1 Flow of Study Participants
