# Supplementary material for: Additional postdilatation using noncompliant balloons after everolimus‐eluting stent implantation: Results of the PRESS trial
Source: Clin Cardiol. 2020 Mar 16;43(6):606–13. doi: 10.1002/clc.23355 (PMC7298980; doi:10.1002/clc.23355)

**Supplementary Figure 1.** Flow of Study Participants

MI = myocardial infarction; TVR = target vessel revascularization

^*^We have no reliable data for patients assessed for eligibility.


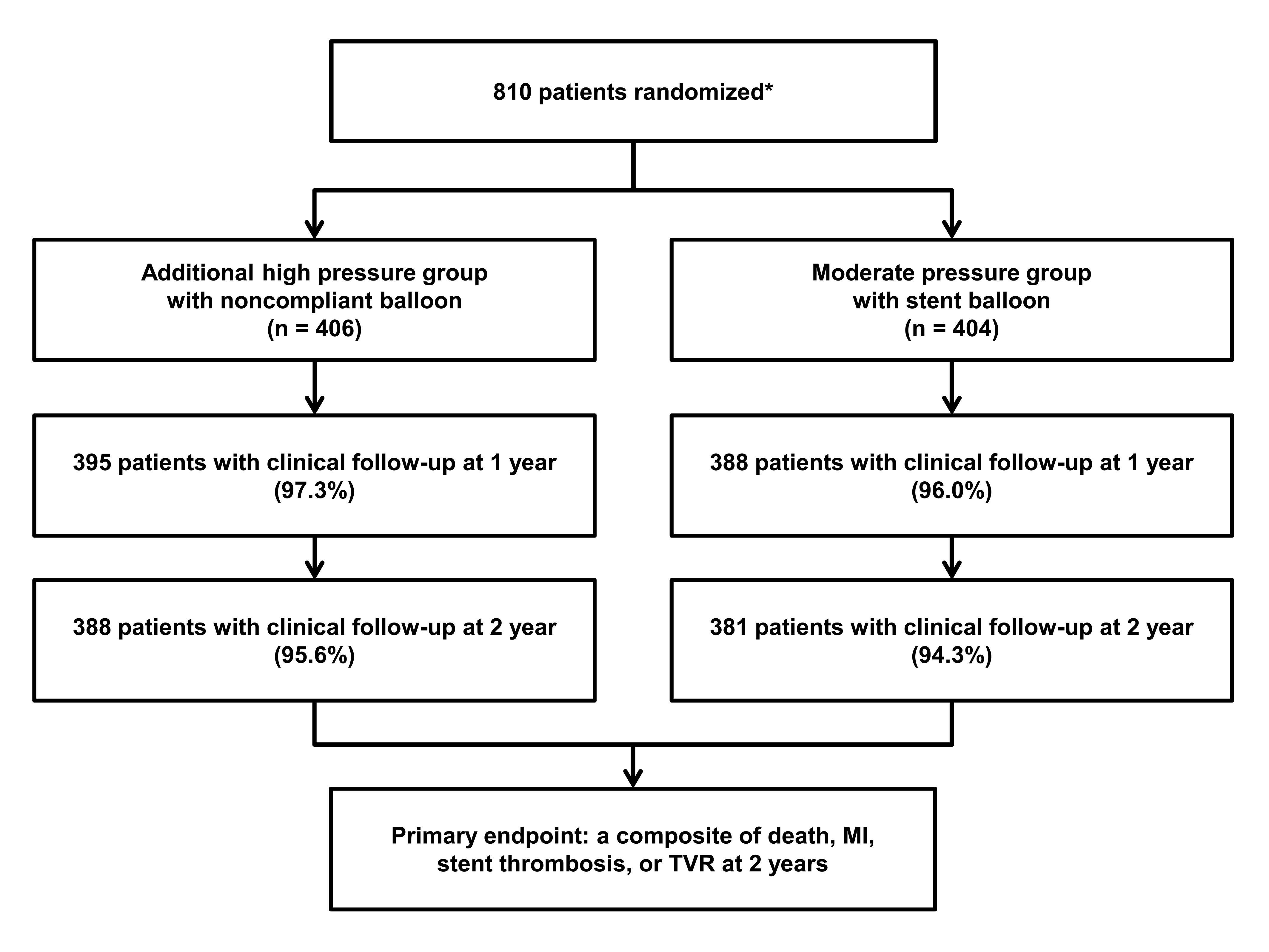

Supplement: Supplementary file 1 — Supplementary Figure 1 Flow of Study Participants [file CLC-43-606-s001.doc]
